# Human and Hunting Dog Interactions in the United States: Exploring Potential Transmission Pathways of Zoonotic Diseases and Highly Pathogenic Avian Influenza Virus

**DOI:** 10.3390/vetsci13050449

**Published:** 2026-05-02

**Authors:** Rachel S. Ziejka, Justin D. Brown, Sally Thompson-Iritani, Vickie Ramirez, Hannah T. Fenelon, Marissa G. Baker

**Affiliations:** 1 Department of Environmental & Occupational Health Sciences, University of Washington, Seattle, WA 98195, USA; rziejka@uw.edu (R.S.Z.); bakermg@uw.edu (M.G.B.); 2 Department of Veterinary and Biomedical Sciences, The Pennsylvania State University, University Park, PA 16802, USA; 3 Department of Environmental & Occupational Health Sciences and Office of Research, University of Washington, Seattle, WA 98195, USA; sti2@uw.edu; 4 Center for One Health Research, Department of Environmental & Occupational Health Sciences, University of Washington, Seattle, WA 98195, USA; ramirezv@uw.edu (V.R.);

**Keywords:** avian influenza, hunting dog, zoonotic disease

## Abstract

Since 2022, numerous H5N1 highly pathogenic avian influenza virus (HPAIV) detections have been reported in wild and domestic mammals in North America. Although H5N1 HPAIV detections in dogs are rare, hunting dogs that retrieve waterfowl are at increased exposure risk due to their physical contact with reservoirs (waterfowl) and contaminated environments. A cross-sectional survey of hunters was conducted during 2024 to characterize hunting procedures, disease prevention practices, and interactions between humans and their hunting dogs to identify potential risks for zoonotic disease transmission. Descriptive analysis (N = 112) indicated a majority of participants considered their hunting dog as part of the family (93.8%), and less than half considered their dog a pet (42.9%). Of the 112 individuals, 96.4% did not utilize personal protective equipment (PPE) when handling a sick dog and 81.3% did not use PPE when handling harvested birds. This research demonstrated complex, sustained physical and personal connections between individuals and their hunting dogs. Additional research utilizing a One Health approach is necessary to define H5N1 HPAIV risk factors in hunting dogs and the environment’s role in the transmission of viruses among wildlife and domestic animals. Understanding zoonotic disease transmission in these populations can inform approaches to mitigate viral exposure.

## Simple Summary

Since 2022, detections of H5N1 highly pathogenic avian influenza virus (HPAIV) in a diversity of wild and domestic mammals in North America have elevated concerns about the impacts of this pathogen on both animal and human health. Hunting dogs that retrieve wild birds are at elevated risk for exposure to a variety of zoonotic diseases. Specifically, dogs that retrieve waterfowl, a known reservoir for H5N1 HPAIV, could be at increased risk for exposure to this virus. In this study, we conducted a cross-sectional survey of hunters that hunt with dogs to characterize hunting practices, disease prevention efforts, and interactions between hunters and their dogs. The results of this study defined a close and complex connection between hunters, their family, and their hunting dogs. In addition, this study identified multiple potential transmission risks. This information helps define risks for H5N1 HPAIV exposure and other zoonotic diseases in hunting dogs and hunters and can serve as a basis for developing strategies to mitigate virus infection in these hosts.

## 1. Introduction

Greater than 60% of infectious diseases known to affect humans spread from animals and approximately 75% of emerging infectious diseases in people are classified as zoonotic diseases [1]. Over the last 20 years, there have been several significant human infectious diseases with documented origins in animals, including severe acute respiratory syndrome (SARS), Ebola, Middle East respiratory syndrome (MERS), Zika, and COVID-19 [2]. Consequently, public health surveillance programs have increasingly incorporated animals into their monitoring efforts, especially wildlife, due to their frequent role as reservoirs for many pathogens and sources of spillover into humans and domestic animals. Hunting dogs, by the nature of their field activities and living in close proximity to their owners, reside at an interface between wildlife, domestic animals, and humans and have a potentially important role in the transmission of diseases between all three sectors. In addition to being sources of disease transmission, hunting dogs may be important sentinels for diseases of public health or veterinary importance.

Since emerging in the late 1990s, the A/goose/Guangdong/1/1996 (Gs/Gd; H5N1) lineage of highly pathogenic avian influenza (HPAIV) has spread globally, impacting the health of domestic animals, wildlife, and humans [3]. In North America, Gs/Gd H5N1 HPAIV subclade 2.3.4.4b has caused morbidity and mortality in wildlife, agricultural animals, companion animals, and humans since it was first identified in late 2021 [4]. The unprecedented duration, global distribution, and diversity of species affected by the current strains of H5N1 HPAIV differ from other strains of HPAIV that historically were strongly adapted to and infect poultry compared to wild birds [5,6]. As of 7 May 2025, in the United States (US), there have been over 13,001 confirmed detections of H5N1 HPAIV in wild birds, with 169,329,161 poultry affected, and 466 confirmed cases in wild mammals [7,8]. With each additional identification of H5N1 HPAIV in a wild or domestic mammal, including canids, there are concerns that the virus is adapting to mammals [8,9,10,11,12,13,14]. The risks to human health have become a greater concern due to the number and diversity of wild and domestic species affected, increasing the potential for exposure, along with poorly defined mechanisms of spillover to humans [15]. The first mammal-to-human transmission of H5N1 HPAIV (dairy cow to human) was reported in Spring 2024 in the US [16] and the first human case with no known exposure also occurred in the US in Fall 2024 [17].

Historically, Gs/Gd H5 HPAIV morbidity or mortality in domestic dogs has been extremely rare [18,19]. When domestic dog infections have been detected, they have involved clinically ill or dead animals with histories of close exposure to infected birds. Although documented natural H5 HPAIV infections in dogs are not common, experimental studies indicate that dogs are susceptible to infection and experience varying disease between viral strains. These studies indicate that dogs can be infected and shed H5N1 HPAIV and may thus represent a potential source of infection for humans [20,21].

Hunting dogs are at an increased risk for various diseases from exposure to a multitude of pathogens through their field activities, including, but not limited to, leptospirosis, tick-borne diseases, and *Cryptosporidium* spp. [22,23]. Combined with their proximity to humans, they may be prime candidates to monitor and assess risks for zoonotic pathogens. Compared to most companion dogs, hunting dogs are more likely to interact closely with wild animals and spend a greater amount of time in the natural environment, potentially increasing their exposure to pathogens harbored by wildlife [22,23,24], especially pathogens with transmission mechanisms that involve the environment, (e.g., avian influenza viruses (AIVs)) [25]. Dogs that hunt may also be exposed to infectious diseases by ingesting infected raw meat in their diet or while game is being processed in the field [23]. Additionally, hunting dogs may travel long distances to hunt specific game in various locations, exposing them to pathogens found in a diversity of habitats and regions beyond just their home environment [23]. All of these mechanisms for pathogen exposure contribute to the increased risk for zoonotic infection in hunting dogs, including H5N1 HPAIV. Specifically, dogs that retrieve waterfowl (e.g., ducks), which can be asymptomatically infected with H5N1 HPAIV, may be at the highest risk for viral exposure, unlike dogs that retrieve upland game birds (e.g., pheasant and quail), which are more likely to experience severe infection and high mortality [26,27]. Waterfowl hunting dogs retrieve downed birds in their mouths from aquatic environments [28]. This category of hunting dogs can be exposed to zoonotic diseases for prolonged periods of time through direct physical contact with wildlife or infected environments [29,30]. Frequent interactions with their human handlers, combined with this extended exposure to potentially infected birds and environments, could enable disease spillover. Although hunting dogs are potentially an important transmission pathway of various zoonotic diseases, including H5N1 HPAIV, to humans, other mechanisms for human exposure may occur through interactions with wildlife, domestic animals, or their environments.

Previous research has considered hunting dogs as sentinels for human health [31,32,33]; however, few studies have evaluated hunting dogs as sources for Gs/Gd H5 HPAIV in humans. Currently, there are no reported cases of dog-to-human transmission of H5N1 HPAIV. However, previous serosurveys have identified antibodies to human influenza viruses in pet dogs, providing evidence that transmission of influenza viruses through close contact between pet and owner is possible [34,35,36]. Thus, it represents prudent public health practice to investigate potential routes of transmission between dogs and humans, given their close companionship and cohabitation; this would inform not only the transmission of H5N1 HPAIV but other zoonotic diseases as well.

Due to the continued and widespread outbreaks of H5N1 HPAIV in the US affecting an increasing number of wild and domestic animals, including cases of mammal-to-human transmission, hunting dogs and their owners appear as an important group to consider when investigating the risk of H5N1 HPAIV spillover to humans or companion animals. Since H5N1 HPAIV infection has been identified in dogs after high levels of exposure, and spillover to humans from dogs has yet to occur, there is the opportunity to consider and implement relatively easy preventative measures to mitigate risks to both dogs and humans. In this study, we aimed to determine hunting dog owners’ contact with and perceptions of their dogs using an online survey. The goal of this research was to define the risks for H5N1 HPAIV transmission between hunting dogs and humans in order to provide foundational knowledge for subsequent studies investigating the transmission and potential prevention of H5N1 HPAIV.

## 2. Materials and Methods

### 2.1. Study Design and Population

**Figure 1:**
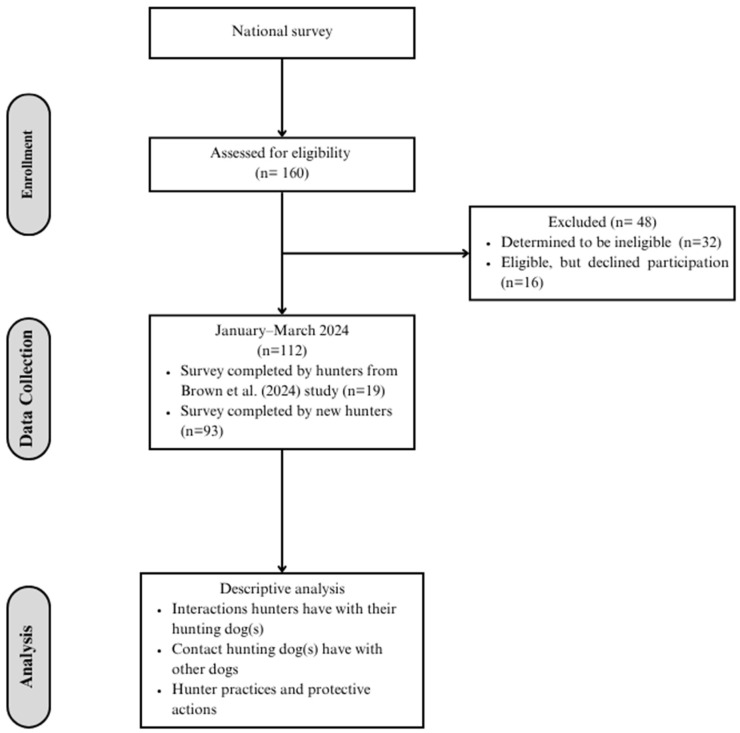
Flow diagram of the study design.

A nationwide survey was used to assess hunter–canine interactions and identify opportunities to introduce preventative measures to mitigate the risk of zoonotic disease transmission between hunting dogs and humans (Figure 1). A group of 112 hunters participated in an online survey that characterized the interactions between themselves and their hunting dogs. The survey population was drawn from a previous population of bird dog hunters studied in Washington State, described by Brown et al. [37] , as well as new hunters from across the US.

Recruitment flyers describing the purpose and goals of the survey were distributed electronically via email to individual hunters, hunting listservs, and social media to recruit participants from January 2024 through March 2024. Participants were also recruited through events hosted by hunting retriever clubs and national organizations with an interest in hunting dogs. Participants were eligible to complete the survey after confirming they had a hunting dog, had hunted birds within the previous 12 months (including hunting, hunt tests, and/or field trials), were fluent in English, were 18 years or older, and were a US resident. There were 48 participants excluded from the study due to either being deemed ineligible based on the requirements of the survey (*n* = 32) or eligible but declining to complete the survey (*n* = 16). All study protocols were reviewed and approved by the University of Washington’s Institutional Review Board (STUDY00000042).

### 2.2. Survey

The online survey was open from January 2024 through March 2024. Study data were collected and managed using REDCap electronic data capture tools hosted at the Institute for Translational Health Sciences at the University of Washington [38,39,40].

To ascertain actions that may increase a person’s risk for pathogen exposure or transmission and determine possible events for animal-to-animal transmission, the survey asked about the types of interactions the owner had with their hunting dog(s), whether personal protective practices were employed when handling a sick dog and during hunting activities, and the types of interactions that their hunting dog(s) had with dogs from outside their household. Survey questions were informed by previous studies conducted by the Center for One Health Research (University of Washington, Seattle, WA, USA) [41], experts in the field, and veterinarians and piloted by individuals (*n* = 5) with knowledge of the field and/or who were active bird hunters with dogs. Revisions to the survey were completed based on feedback from the pilot group. The full survey is available in Supplementary Materials.

### 2.3. Data Analysis

Data was cleaned and analyzed using R (version 4.2.2) [42]. Descriptive statistics were computed to assess the frequency of survey respondents’ interactions with their hunting dogs and their dogs’ contact with other dogs outside the household.

## 3. Results

The survey elicited 112 complete responses over 11 weeks. Characteristics of the study population are provided in Table 1.

Hunters from 24 states were represented, with the majority of respondents from Washington State (25.0%) and California (23.2%). Descriptive statistics for how respondents perceived their hunting dogs and locations where they had contact with their dogs are presented in Table 2. The majority of respondents indicated that they considered their dog a part of the family (93.8%) and 42.9% considered their dog a pet.

When participants were asked to consider their response to a dog displaying signs of illness, the most common practice was to keep the dog at home to monitor them, while few other protective measures were implemented (Table 3).

When considering contact between individuals, hunting dogs, and birds, 91 respondents (81.2%) did not use PPE when handling birds in the field (Table 4). The majority of individuals (78.6%) did not use any type of PPE when handling either a dog that displayed signs of illness or birds in the field. In this study, 15.2% of participants reported experiencing undiagnosed flu-like symptoms such as fever, chills, coughing, or trouble breathing within the past year. Information regarding the types of contact a participant’s dog had with dogs outside their immediate household is also exhibited in Table 4. Most respondents (73.0%) indicated that their dog had contact with both non-hunting and hunting dogs outside their household.

## 4. Discussion

This study of hunting dog interactions with humans and other dogs provides valuable insight into a population at potentially increased risk for infections with zoonotic influenza viruses such as H5N1 HPAIV. Results from this study reveal that nearly half of participants consider their hunting dog a pet and/or part of the family, rarely use PPE when a dog is sick, and report frequent contact between hunting dogs and other dogs, providing critical knowledge of instances where disease transmission could occur, including dog-to-human and dog-to-dog transmissions.

A majority of respondents reported using no PPE when handling harvested birds or interacting with a sick dog. While few individuals reported experiencing any undiagnosed flu-like symptoms, we should still be cautious about the potential for infectious diseases to move between humans and animals. Although the public health risk remains low for H5N1 HPAIV according to the CDC [7], the increasing number and diversity of mammal cases raises concern that the virus is adapting to various mammal species, making work like this important should dog-to-human transmission occur in the future. Despite the reportedly low threat to the public’s health, preventive precautions are still warranted. The use of basic PPE is an easy, cost-efficient approach to assist in preventing some disease transmission between humans and animals. In response to the H5N1 HPAIV outbreak, there have been recommendations for waterfowl hunters to utilize PPE such as gloves, eyewear, and masks to prevent infection [43,44,45,46], especially since commonly hunted waterfowl such as ducks may be infected but display no outward signs of disease. However, limited literature exists describing PPE usage among hunters, and our findings similarly indicate low utilization of PPE. Gloves or eyewear may be used to prevent contact with infected bodily fluids and specimens. Utilizing masks during activities such as processing birds or treating a sick dog can reduce the risk of inhaling infectious aerosols. Field dressing game in a well-ventilated area is also a recommendation that can limit aerosol exposure. Developing a plan to disinfect and contain potentially contaminated materials (e.g., clothes and equipment) after use in a high-risk activity, such as hunting waterfowl, may also prevent unnecessary prolonged exposure to infectious materials and decrease the exposure of other humans and animals to disease. Although PPE use may be the easiest control method to employ, it is also considered the least effective hazard control strategy in the hierarchy of controls, as PPE only protects the individual user and is only effective if the person consistently utilizes it properly; similarly, it would not prevent animal-to-animal transmission [47]. Implementing other forms of controls, such as engineering and administrative controls that address biosafety measures alongside PPE, can further increase the effectiveness of risk management strategies while reducing the risk of disease transmission to the public. Although some of the risks and mitigation strategies highlighted in this study may be applicable to multiple zoonotic pathogens, additional studies are necessary to evaluate the specific risks and prevention efforts for other pathogens that can be transmitted between hunting dogs and humans. Additional research would also be needed to standardize zoonotic disease risk mitigation practices within the human and veterinary fields. Basic biosafety, including wearing appropriate PPE, cleaning and disinfecting equipment and living spaces, and processing birds in well-ventilated areas, is essential to developing future risk mitigation protocols.

Besides PPE use, individuals with hunting dogs may consider initiating an open dialogue with both their human health provider and veterinarian to discuss the zoonotic disease risks , such as H5N1 HPAIV, and develop preventative measures to mitigate risks where possible. For instance, 26.8% of participants reported feeding parts of the birds they harvested to their dogs. Conversations with their veterinarian about feeding uncooked bird parts and allowing dogs to scavenge dropped pieces during processing will help identify possible pathogen exposure risks to hunting dogs, including H5N1 HPAIV-infected meat. Hunters may also discuss the potential for H5N1 HPAIV infection with their doctor if harvested meat is not cooked to an internal temperature of 165 °F, which inactivates H5N1 HPAIV within or on the meat [48]. Together, a plan including PPE, biosafety, monitoring their hunting dog for clinical signs of disease, vaccinations (where applicable), and engaging a health professional before or during illness in either their dog or themselves can be developed to reduce the risk of disease transmission from the field while hunting to people and animals at home. Other similar conversations could be undertaken for general zoonotic diseases, discussing how interactions with dogs could lead to increased risk of transmission of disease through inhalation, ingestion, and dermal routes of exposure.

Hunting organizations and licensing departments could also consider providing educational materials and training events to hunters about circulating or emerging zoonotic diseases, especially those with hunting dogs, regarding disease exposure, transmission, and proper preventative actions. These groups can provide basic information about protective measures for current outbreaks, such as H5N1 HPAIV. For example, although some waterfowl species may not display outward signs of H5N1 HPAIV infection, any bird exhibiting signs of illness or abnormal behavior or sites with multiple sick or dead birds present should be avoided during hunting activities and reported to the proper regulatory agency. Utilizing the data generated from this study about the risks for H5N1 HPAIV transmission, combined with the resources of hunting organizations and management departments, procedures for mitigating the risk of transmission of zoonotic diseases can be designed specifically for hunting events. Mitigation plans for hunting events may consider strategies to reduce the risk of pathogen transmission at the individual/dog level or at a larger, population or group level. Implementing different levels of control and preventative measures can reduce risks for potential H5N1 HPAIV infection but are also applicable to other infectious diseases that are harbored in wildlife.

In order to develop comprehensive risk mitigation plans to reduce the spread of H5N1 HPAIV and other zoonotic diseases, a One Health approach should be considered. Since 2021, H5N1 HPAIV has continued to expand its host range and caused negative impacts on the health of humans, wildlife, agricultural animals, and companion animals. One Health is an integrated approach that recognizes the interconnectedness of humans, animals, and the environment, including ecosystems, to address a wide range of health issues and generate holistic solutions [49]. In this work, we investigated human and animal populations, but this study did not include any environmental sampling. However, previous studies have demonstrated that the environment acts as an important risk factor for H5N1 HPAIV [29,30]. Climate change is a major environmental element that may impact the epidemiology of H5N1 HPAIV through changes in environmental conditions and bird biology and movements. Increasing temperatures may alter the survival and viability of H5N1 HPAIV in aquatic habitats [50]. Furthermore, changes in temperature and seasonality may affect the abundance, migratory patterns, and geographic range of various bird species [51,52,53] that act as reservoirs for H5N1 HPAIV. Future studies may consider incorporating environmental sampling to screen for the presence of H5N1 HPAIV, along with weather data, to identify possible associations or trends between climate indicators and H5N1 HPAIV. Collecting environmental, animal, and human samples from a shared location may provide an opportunity for disease surveillance.

Besides not including environmental sampling, results from the survey may not be generalizable due to the small sample size. According to a report by the United States Fish and Wildlife Service, there were approximately 1.3 million waterfowl hunters in the US who purchased state hunting licenses in the 2023 hunting season [54]. Furthermore, many of the participants were from either Washington State or California and may not be representative of all hunters in the US. In addition, the survey relied on self-reporting and the questions may not accurately assess the range of experiences of hunters, though we worked with bird dog hunters to develop and pilot the survey and ensure it was appropriate to our population of interest. Furthermore, an atypical outbreak of canine infectious respiratory disease complex (CIRDC) appeared in Oregon in August 2023 and spread across multiple states [55], which may have affected participants’ travel to other states or hunting events and the contact permitted between hunting dogs and other dogs. This survey did not include questions about other potential risk behaviors for hunters, such as consuming food or smoking while hunting, or for dogs, such as coprophagia of bird feces. The frequency of hunting waterfowl would also be an important determinant of exposure risk for hunters but was not characterized in our survey. Frequency may vary by local regulations and individual hunters. Blood samples were not collected from either hunters or their dogs at the time of this survey; therefore, we could not confirm any cases of infection with H5N1 HPAIV or other zoonotic diseases or develop associations between specific behaviors, frequency of hunting events, and the risk of disease.

Overall, examining hunting dogs as potential sentinels of H5N1 HPAIV provides us with the opportunity to determine the unique risks to the health of these dogs, who share our living space and act as links between humans and wildlife reservoirs, and allows for public health preparedness should dog-to-human transmission be a route of H5N1 HPAIV transmission. The contact humans maintain with their hunting dogs support the concern for the potential of disease transmission between humans and animals and the importance of implementing protective practices to reduce disease risk and prevent spillover. Characterizing the interactions humans have with their hunting dogs provides an increased understanding of routes for the virus to enter the human population or for the introduction of human diseases to dogs. Findings from this research suggest the need for continued surveillance of hunting dogs and investigation to better understand the risk factors for hunting dogs becoming infected with H5N1 HPAIV. By leveraging the desire to preserve the health of hunting dogs, we may simultaneously safeguard public health. This study provides the foundation for future research into understanding opportunities for not only the transmission of H5N1 HPAIV but also other infectious diseases between humans and dogs that are both companions and coworkers. Since H5N1 HPAIV is not currently a human pandemic, this enables us to achieve prevention of the disease rather than a response to a public health emergency. Finally, this research provides the basis for future studies seeking to understand the intricate relationship between hunting dogs and their owners and the intermediate connection of domestic animals in the potential spread of infectious diseases between humans and animals.

## Figures and Tables

**Table 1:** Characteristics of study population (N = 112).

	*n* (%)
**State** **of Residence**	
Washington	28 (25.0%)
California	26 (23.2%)
Michigan	9 (8.0%)
Wisconsin	8 (7.1%)
Minnesota	6 (5.4%)
Texas	5 (4.5%)
Oregon	5 (4.5%)
Other ^†^	25 (22.3%)
**Number of Dogs Owned**	
Mean (SD ^‡^)	2.32 (1.57)
Median [IQR ^§^]	2 (1, 3)
**Hunting Activity**	
Hunting	54 (48.2%)
Hunt Tests/Trials	9 (8.0%)
Both	49 (43.8%)
**Participated in AIV**^¶^ **Serosurveillance study**	19 (17.0%)
**Dog CIV ^a^ Vaccinated**	
Yes	43 (38.4%)
No	51 (45.5%)
Do Not Know	18 (16.1%)
**Has Pet Insurance**	37 (33.0%)

^†^ Other states include Colorado, Florida, Georgia, Idaho, Illinois, Indiana, Louisiana, Maryland, Massachusetts, Missouri, Montana, Nevada, New York, North Carolina, Ohio, Pennsylvania, and South Dakota. ^‡^ SD: Standard Deviation. ^§^ IQR: Interquartile Range. ^¶^ AIV: Avian Influenza Virus. ^a^ CIV: Canine Influenza Virus. Headings related to questions asked in the survey appear in bold.

**Table 2:** Hunter perceptions and contact with their hunting dogs (N = 112) ^†^.

	*n* (%)
**I consider my dog:** ^‡^	
Part of the family	105 (93.8%)
Working dog	55 (49.1%)
Tool for hunting	51 (45.5%)
Pet	48 (42.9%)
**Dog lives:**	
Inside	63 (56.2%)
Inside/Outside	47 (42.0%)
Outside	2 (1.8%)
**Of dogs that live inside (*n* = 110), the dog sleeps:**	
In the home	101 (91.8%)
Indoors, not in the home	8 (7.3%)
Outside	1 (0.9%)
**Of dogs that sleep in the home (*n* = 101), the dog sleeps in my room**	83 (83.0%)
**Of dogs that sleep in my room (*n* = 83), the dog sleeps in my bed**	52 (62.7%)
**Kiss my dog**	67 (59.8%)

^†^ Responses may not equal totals since some human–canine contact may not have been applicable. ^‡^ Respondents were asked to select all that applied. Headings related to questions asked in the survey appear in bold.

**Table 3:** Hunters’ responses to dogs who display signs of illness (total N = 112) ^†^.

If My Dog Has Shown Signs of Illness, I ^‡^:	*n* (%)
**Keep at home to monitor**	56 (50%)
**Wash hands**	31 (27.7%)
**Disinfect dog’s living space**	30 (26.8%)
**Separate from other animals**	21 (18.8%)
**Disinfect training equipment or dog gear**	20 (17.9%)
**Wear PPE ^§^**	4 (3.6%)
**Other**	4 (3.6%)
**Separate self from dog**	1 (0.9%)
**Dog has never shown signs of illness**	47 (42.0%)

^†^ Respondents were asked to select all that applied. ^‡^ Signs of illness include diarrhea, lethargy, and lack of appetite. ^§^ PPE: Personal protective equipment. Heading related to question asked in the survey appears in bold.

**Table 4:** Reported contact between hunting dogs and humans, birds, and dogs outside the household (N = 112).

Do You:	*n* (%)
**Process birds in the field**	
Yes	9 (8.0%)
Sometimes	20 (17.9%)
No	83 (74.1%)
**Feed parts of bird to dog**	30 (26.8%)
**Use PPE** ^†^ **use when handling birds**	21 (18.8%)
**Where do you board your dog?**	
General boarding	16 (14.3%)
Boarding at a training facility ^‡^	23 (20.7%)
Do not board dog ^‡^	80 (72.1%)
**Any contact with non-hunting dogs**	84 (75.7%)
**Frequency of contact with non-hunting dogs**	
Daily	7 (8.3%)
Weekly	28 (33.3%)
Monthly	34 (40.5%)
Yearly	15 (17.9%)
**Any contact with other hunting dogs**	100 (89.3%)
**Frequency of contact with other hunting dogs**	
Daily	6 (6.0%)
Weekly	40 (40.0%)
Monthly	44 (44.0%)
Yearly	10 (10.0%)

^†^ PPE: Personal protective equipment. ^‡^ Missing one response. Headings related to questions asked in the survey appear in bold.

## Data Availability

The data presented in this study are openly available in Dryad at https://doi.org/10.5061/dryad.2jm63xt2w.

## Supplementary Materials

The following supporting information can be downloaded at: https://www.mdpi.com/article/10.3390/vetsci13050449/s1.

## Abbreviations

The following abbreviations are used in this manuscript:

HPAIV	Highly pathogenic avian influenza virus
PPE	Personal protective equipment
SARS	Severe acute respiratory syndrome
MERS	Middle East respiratory syndrome
US	United States
CIRDC	Canine infectious respiratory disease complex

## References

[1] (2023). One Health. World Health Organization.

[2] Sharan M., Vijay D., Yadav J.P., Bedi J.S., Dhaka P. (2023). Surveillance and Response Strategies for Zoonotic Diseases: A Comprehensive Review. Sci. One Health.

[3] Mukhtar M.M., Rasool S.T., Song D., Zhu C., Hao Q., Zhu Y., Wu J. (2007). Origin of Highly Pathogenic H5N1 Avian Influenza Virus in China and Genetic Characterization of Donor and Recipient Viruses. J. Gen. Virol..

[4] Spackman E., Pantin-Jackwood M.J., Lee S.A., Prosser D. (2023). The Pathogenesis of a 2022 North American Highly Pathogenic Clade 2.3.4.4b H5N1 Avian Influenza Virus in Mallards (*Anas platyrhynchos*). Avian Pathol..

[5] El-Bidawy M.H., Mohammad I., Ansari M.R., Hajelbashir M.I., Khan M.S., Poyil M.M., Bari M.N., Arafah A.M.R., Kamal M.A., Ahsan S.T.M. (2025). Highly Pathogenic Avian Influenza: Tracking the Progression from IAV (H5N1) to IAV (H7N9) and Preparing for Emerging Challenges. Microorganisms.

[6] U.S. Geological Survey Avian Influenza Spread, Prevalence and Persistence. https://www.usgs.gov/centers/eesc/science/avian-influenza-spread-prevalence-and-persistence.

[7] CDC H5 Bird Flu: Current Situation. https://www.cdc.gov/bird-flu/situation-summary/index.html.

[8] USDA Animal and Plant Health Inspection Service Detections of Highly Pathogenic Avian Influenza in Mammals. https://www.aphis.usda.gov/livestock-poultry-disease/avian/avian-influenza/hpai-detections/mammals.

[9] Burrough E.R., Magstadt D.R., Petersen B., Timmermans S.J., Gauger P.C., Zhang J., Siepker C., Mainenti M., Li G., Thompson A.C. (2024). Highly Pathogenic Avian Influenza A(H5N1) Clade 2.3.4.4b Virus Infection in Domestic Dairy Cattle and Cats, United States, 2024. Emerg. Infect. Dis..

[10] Elsmo E.J., Wünschmann A., Beckmen K.B., Broughton-Neiswanger L.E., Buckles E.L., Ellis J., Fitzgerald S.D., Gerlach R., Hawkins S., Ip H.S. (2023). Highly Pathogenic Avian Influenza A(H5N1) Virus Clade 2.3.4.4b Infections in Wild Terrestrial Mammals, United States, 2022. Emerg. Infect. Dis..

[11] Niedringhaus K.D., Chan T.C., McDowell A., Maxwell L., Stevens M., Potts L., Miller E., Anis E., Why K.V., Keller T. (2024). Surveillance of Highly Pathogenic Avian Influenza Virus in Wild Canids from Pennsylvania, USA. Animals.

[12] Plaza P.I., Gamarra-Toledo V., Euguí J.R., Lambertucci S.A. (2024). Recent Changes in Patterns of Mammal Infection with Highly Pathogenic Avian Influenza A(H5N1) Virus Worldwide. Emerg. Infect. Dis..

[13] Reperant L.A., van Amerongen G., van de Bildt M.W.G., Rimmelzwaan G.F., Dobson A.P., Osterhaus A.D.M.E., Kuiken T. (2008). Highly Pathogenic Avian Influenza Virus (H5N1) Infection in Red Foxes Fed Infected Bird Carcasses. Emerg. Infect. Dis..

[14] Peacock T.P., Moncla L., Dudas G., VanInsberghe D., Sukhova K., Lloyd-Smith J.O., Worobey M., Lowen A.C., Nelson M.I. (2025). The Global H5N1 Influenza Panzootic in Mammals. Nature.

[15] Runstadler J.A., Puryear W.B. (2024). The Virus Is out of the Barn: The Emergence of HPAI as a Pathogen of Avian and Mammalian Wildlife around the Globe. Am. J. Vet. Res..

[16] CDC Current Situation: Bird Flu in Dairy Cows. https://www.cdc.gov/bird-flu/situation-summary/mammals.html.

[17] CDC CDC Confirms Human H5 Bird Flu Case in Missouri. https://www.cdc.gov/media/releases/2024/s0906-birdflu-case-missouri.html.

[18] Canadian Food Inspection Agency Domestic Dog Tests Positive for Avian Influenza in Canada. https://www.canada.ca/en/food-inspection-agency/news/2023/04/domestic-dog-tests-positive-for-avian-influenza-in-canada.html.

[19] Songserm T., Amonsin A., Jam-on R., Sae-Heng N., Pariyothorn N., Payungporn S., Theamboonlers A., Chutinimitkul S., Thanawongnuwech R., Poovorawan Y. (2006). Fatal Avian Influenza A H5N1 in a Dog. Emerg. Infect. Dis..

[20] Maas R., Tacken M., Ruuls L., Koch G., van Rooij E., Stockhofe-Zurwieden N. (2007). Avian Influenza (H5N1) Susceptibility and Receptors in Dogs. Emerg. Infect. Dis..

[21] Chen Y., Zhong G., Wang G., Deng G., Li Y., Shi J., Zhang Z., Guan Y., Jiang Y., Bu Z. (2010). Dogs Are Highly Susceptible to H5N1 Avian Influenza Virus. Virology.

[22] Sgroi G., Buono F., Iatta R., Beall M., Chandrashekar R., Buch J., Piantedosi D., Veneziano V., Otranto D. (2022). Vector-Borne Pathogens of Zoonotic Concern in Hunting Dogs of Southern Italy. Acta Trop..

[23] Ridgway M. (2021). Hunting Dogs. Vet. Clin. Small Anim. Pract..

[24] Cilia G., Fratini F., Turchi B., Ebani V.V., Turini L., Bilei S., Bossù T., De Marchis M.L., Cerri D., Bertelloni F. (2021). Presence and Characterization of Zoonotic Bacterial Pathogens in Wild Boar Hunting Dogs (*Canis lupus familiaris*) in Tuscany (Italy). Animals.

[25] Stallknecht D.E., Brown J.D. (2009). Tenacity of Avian Influenza Viruses: -EN- Tenacity of Avian Influenza Viruses -FR- La Stabilité Des Virus de l’influenza Aviaire -ES- Persistencia de Los Virus de La Influenza Aviar. Rev. Sci. Tech. OIE.

[26] Food and Agriculture Organization of the United Nations (2026). FAO Alert on Avian Influenza—Risk of Upsurge and Regional Spread through Wild Birds in Latin America and the Caribbean.

[27] Crespo R., França M.S., Fenton H., Shivaprasad H.L. (2018). Galliformes and Columbiformes. Pathology of Wildlife and Zoo Animals.

[28] WDFW Hunter Education Division (2019). The Basics of Waterfowl Hunting in Washington. https://wdfw.wa.gov/sites/default/files/2020-09/basics_of_waterfowl_hunting.pdf.

[29] Dovas C.I., Papanastassopoulou M., Georgiadis M.P., Chatzinasiou E., Maliogka V.I., Georgiades G.K. (2010). Detection and Quantification of Infectious Avian Influenza A (H5N1) Virus in Environmental Water By Using Real-Time Reverse Transcription-PCR. Appl. Environ. Microbiol..

[30] Horm S.V., Gutiérrez R.A., Sorn S., Buchy P. (2012). Environment: A Potential Source of Animal and Human Infection with Influenza A (H5N1) Virus. Influenza Other Respir. Viruses.

[31] Gómez-Morales M.A., Selmi M., Ludovisi A., Amati M., Fiorentino E., Breviglieri L., Poglayen G., Pozio E. (2016). Hunting Dogs as Sentinel Animals for Monitoring Infections with *Trichinella* spp. in Wildlife. Parasites Vectors.

[32] Machado F.P., Kmetiuk L.B., Teider-Junior P.I., Pellizzaro M., Yamakawa A.C., Martins C.M., Bach R.v.W., Morikawa V.M., de Barros-Filho I.R., Langoni H. (2019). Seroprevalence of Anti-Toxoplasma Gondii Antibodies in Wild Boars (*Sus. scrofa*), Hunting Dogs, and Hunters of Brazil. PLoS ONE.

[33] Mahachi K., Kontowicz E., Anderson B., Toepp A.J., Lima A.L., Larson M., Wilson G., Grinnage-Pulley T., Bennett C., Ozanne M. (2020). Predominant Risk Factors for Tick-Borne Co-Infections in Hunting Dogs from the USA. Parasites Vectors.

[34] Dundon W.G., De Benedictis P., Viale E., Capua I. (2010). Serologic Evidence of Pandemic (H1N1) 2009 Infection in Dogs, Italy. Emerg. Infect. Dis..

[35] Jang H., Jackson Y.K., Daniels J.B., Ali A., Kang K., Elaish M., Lee C.-W. (2017). Seroprevalence of Three Influenza A Viruses (H1N1, H3N2, and H3N8) in Pet Dogs Presented to a Veterinary Hospital in Ohio. J. Vet. Sci..

[36] Sooksawasdi Na Ayudhya S., Kuiken T. (2021). Reverse Zoonosis of COVID-19: Lessons from the 2009 Influenza Pandemic. Vet. Pathol..

[37] Brown J.D., Black A., Haman K.H., Diel D.G., Ramirez V.E., Ziejka R.S., Fenelon H.T., Rabinowitz P.M., Stevens L., Poulson R. (2024). Antibodies to Influenza A(H5N1) Virus in Hunting Dogs Retrieving Wild Fowl, Washington, USA. Emerg. Infect. Dis..

[38] Harris P.A., Taylor R., Thielke R., Payne J., Gonzalez N., Conde J.G. (2009). Research Electronic Data Capture (REDCap)—A Metadata-Driven Methodology and Workflow Process for Providing Translational Research Informatics Support. J. Biomed. Inf..

[39] Harris P.A., Taylor R., Minor B.L., Elliott V., Fernandez M., O’Neal L., McLeod L., Delacqua G., Delacqua F., Kirby J. (2019). The REDCap Consortium: Building an International Community of Software Platform Partners. J. Biomed. Inf..

[40] Harris P.A., Delacqua G., Taylor R., Pearson S., Fernandez M., Duda S.N. (2021). The REDCap Mobile Application: A Data Collection Platform for Research in Regions or Situations with Internet Scarcity. JAMIA Open.

[41] Meisner J., Baszler T.V., Kuehl K.E., Ramirez V., Baines A., Frisbie L.A., Lofgren E.T., de Avila D.M., Wolking R.M., Bradway D.S. (2022). Household Transmission of SARS-CoV-2 from Humans to Pets, Washington and Idaho, USA. Emerg. Infect. Dis..

[42] R Core Team (2022). R: A Language and Environment for Statistical Computing.

[43] CDC Hunters and Bird Flu. https://www.cdc.gov/bird-flu/risk-factors/hunters-and-bird-flu.html.

[44] Stop the Spread of Bird Flu: Public Asked Not to Touch Wild Birds and to Take Precautions Handling Domestic Birds. https://agr.wa.gov/about-wsda/news-and-media-relations/news-releases?article=45182.

[45] Colaianni M. Caution Urged During Avian Flu Outbreak. http://www.azgfd.com/2025/01/08/azgfd-issues-precautions-during-avian-influenza-outbreak/.

[46] Michigan Department of Natural Resources HPAI FAQs. https://www.michigan.gov/dnr/managing-resources/wildlife/wildlife-disease/disease-monitoring/avian-influenza-updates/hpai-faqs.

[47] CDC|NIOSH Hierarchy of Controls. https://www.cdc.gov/niosh/topics/hierarchy/default.html.

[48] Swayne D.E. (2016). Animal Influenza.

[49] World Health Organization One Health. https://www.who.int/health-topics/one-health.

[50] Brown J., Goekjian G., Poulson R., Valeika S., Stallknecht D. (2009). Avian Influenza Virus in Water: Infectivity Is Dependent on pH, Salinity and Temperature. Vet. Microbiol..

[51] Horton K.G., La Sorte F.A., Sheldon D., Lin T.-Y., Winner K., Bernstein G., Maji S., Hochachka W.M., Farnsworth A. (2020). Phenology of Nocturnal Avian Migration Has Shifted at the Continental Scale. Nat. Clim. Chang..

[52] Saino N., Ambrosini R., Rubolini D., von Hardenberg J., Provenzale A., Hüppop K., Hüppop O., Lehikoinen A., Lehikoinen E., Rainio K. (2010). Climate Warming, Ecological Mismatch at Arrival and Population Decline in Migratory Birds. Proc. R. Soc. B Biol. Sci..

[53] Socolar J.B., Epanchin P.N., Beissinger S.R., Tingley M.W. (2017). Phenological Shifts Conserve Thermal Niches in North American Birds and Reshape Expectations for Climate-Driven Range Shifts. Proc. Natl. Acad. Sci. USA.

[54] Raftovich R.V., Fleming K.K., Chandler S.C., Cain C.M. (2024). Migratory Bird Hunting Activity and Harvest During the 2022–2023 and 2023–2024 Hunting Seasons.

[55] American Veterinary Medical Association Questions Remain as Canine Respiratory Disease Cases Fall. https://www.avma.org/news/questions-remain-canine-respiratory-disease-cases-fall.

