# Supplementary material for: Human and Hunting Dog Interactions in the United States: Exploring Potential Transmission Pathways of Zoonotic Diseases and Highly Pathogenic Avian Influenza Virus
Source: Vet Sci. 2026 May 2;13(5):449. doi: 10.3390/vetsci13050449 (PMC13211554; doi:10.3390/vetsci13050449)
Supplement: Supplementary file 1 [file vetsci-13-00449-s001.zip › vetsci-4228779-supplementary.pdf]

National survey completed by hunters to assess the interactions between humans and hunting dogs.

Hunting Dog Interactions

As a graduate student at the University of Washington with a background in animal behavior and wildlife conservation, I am interested in identifying events where human-wildlife contact may occur along with the associated health impacts for both animals and humans. These questions will provide a better understanding of indirect human-wildlife interactions, mediated by hunting dogs. Understanding the connection and contact you have with your dog may enable us to determine different factors that influence the risk of exposure to disease for both your dog as well as yourself along with opportunities for prevention of disease transmission. Your insight and participation are greatly appreciated.

When answering these questions, please consider all of your hunting dogs.

Name

____

What state do you live in?

- ◯ Alabama
- ◯ Alaska
- ◯ Arizona
- ◯ Arkansas
- ◯ California
- ◯ Colorado
- ◯ Connecticut
- ◯ Delaware
- ◯ Florida
- ◯ Georgia
- ◯ Hawaii
- ◯ Idaho
- ◯ Illinois
- ◯ Indiana
- ◯ Iowa
- ◯ Kansas
- ◯ Kentucky
- ◯ Louisiana
- ◯ Maine
- ◯ Maryland
- ◯ Massachusetts
- ◯ Michigan
- ◯ Minnesota
- ◯ Mississippi
- ◯ Missouri
- ◯ Montana
- ◯ Nebraska
- ◯ Nevada
- ◯ New Hampshire
- ◯ New Jersey
- ◯ New Mexico
- ◯ New York
- ◯ North Carolina
- ◯ North Dakota
- ◯ Ohio
- ◯ Oklahoma
- ◯ Oregon
- ◯ Pennsylvania
- ◯ Rhode Island
- ◯ South Carolina
- ◯ South Dakota
- ◯ Tennessee
- ◯ Texas
- ◯ Utah
- ◯ Vermont
- ◯ Virginia
- ◯ Washington
- ◯ West Virginia
- ◯ Wisconsin
- ◯ Wyoming

Did you participate in the Surveillance for Antibodies to H5 Avian Influenza Virus in Hunting Dogs study?

- ◯ Yes
- ◯ No

Which of these do you and your dog(s) participate in?

- ◯ Hunting
- ◯ Hunt tests or trials
- ◯ Both

How many dogs do you have (both hunting and non-hunting dogs)?

____

______

Do your dog(s) live indoor, outdoor, or indoor/outdoor? Check all that apply.	☐ Indoor<br>☐ Indoor/Outdoor<br>☐ Outdoor
Where do your dog(s) sleep at night?	○ In the home<br>○ Indoors, not in the home (e.g. barn, kennel)<br>○ Outside
Do your dog(s) sleep in your room?	○ Yes ○ No
Do the dog(s) sleep in your bed?	○ Yes ○ No
I consider my dog(s):<br>Check all that apply.	☐ Part of the family<br>☐ A pet<br>☐ A working dog<br>☐ A tool for hunting
How do you transport your dog(s) to different locations?	○ Inside the vehicle with you (e.g. backseat, passenger seat) ○ Separate from you (e.g. trailer, pick-up bed)
If your dog(s) have shown any signs of illness (e.g. diarrhea, lethargy, lack of appetite), have you ever done any of the following? Check all that apply.	☐ Kept them in the home to monitor them<br>☐ Handwashing after interacting with potentially sick dog<br>☐ Separate dog from other animal<br>☐ Separate yourself from the dog (e.g. put the dog in isolation, do not bring into the house)<br>☐ Disinfect area where dog lives<br>☐ Disinfect training equipment or dog gear<br>☐ Wear PPE (personal protective equipment) when working with the dogs (e.g. gloves, mask, etc.)<br>☐ Other<br>☐ My dog has never shown any of these signs of illness
What type of PPE do you wear? Check all that apply.	☐ Gloves<br>☐ Mask<br>☐ Respirator<br>☐ Other
Please list or describe other PPE used.	____
Please list or describe any other precautions you take if your dog shows any signs of illness.	____
Do you process the birds in the field a majority of the time (leaving any parts of the bird in the field)?	○ Yes ○ Sometimes<br>○ No
Do you feed your dog(s) parts from the animals they retrieve?	○ Yes ○ No
Do you wear PPE (personal protective equipment) when handling birds (e.g. mask, gloves, etc.) in the field?	○ Yes ○ No

What do you wear? Check all that apply.	☐ Gloves<br>☐ Mask<br>☐ Eyewear<br>☐ Other
Please list or describe any other PPE that you wear when handling the birds in the field.	______________________________
Did you board your dog(s) at a facility (e.g. daycare) within the last 12 months?	◯ Yes ◯ No
Did you board your dog(s) at a training facility within the last 12 months?	◯ Yes ◯ No
Have your dog(s) been in contact with any non-hunting dogs outside of your household within the last 12 months?	◯ Yes ◯ No
How often on average are your dog(s) in contact with these non-hunting dogs?	◯ Daily<br>◯ Weekly<br>◯ Monthly<br>◯ Yearly
Have your dog(s) been in contact with any hunting dogs outside of your household within the last 12 months?	◯ Yes ◯ No
How often on average are your dog(s) in contact with these hunting dogs?	◯ Daily<br>◯ Weekly<br>◯ Monthly<br>◯ Yearly
Do you have pet insurance for your dog(s)?	◯ Yes ◯ No
Did your dog(s) receive a canine influenza virus (CIV) vaccination in 2023?	◯ Yes<br>◯ No<br>◯ Do not know
Do you kiss your dog?	◯ Yes ◯ No
In the last 12 months, have YOU experienced any undiagnosed flu-like symptoms (e.g. fever, chills, coughing, trouble breathing)?	◯ Yes ◯ No
Would you like to be contacted for future studies?	◯ Yes<br>◯ No
Please enter your email address.	______________________________
